# Desmopressin Acetate in Intracranial Haemorrhage

**DOI:** 10.1155/2014/298767

**Published:** 2014-12-23

**Authors:** Thomas Kapapa, Stefan Röhrer, Sabine Struve, Matthias Petscher, Ralph König, Christian Rainer Wirtz, Dieter Woischneck

**Affiliations:** ^1^Neurochirurgische Klinik, Universitätsklinikum Ulm, Albert-Einstein-Allee 23, 89081 Ulm, Germany; ^2^Klinik für Hämatologie, Onkologie, Immunologie, Palliativmedizin, Infektiologie und Tropenmedizin, Städtisches Klinikum München, Klinikum Schwabing, Kölner Platz 1, 80804 München, Germany; ^3^Klinik für Anästhesiologie, Abteilung Klinische Anästhesiologie, Albert-Einstein-Allee 23, 89081 Ulm, Germany; ^4^Neurochirurgische Klinik, Klinikum Landshut, Robert-Koch-Strasse 1, 84034 Landshut, Germany

## Abstract

*Introduction*. The secondary increase in the size of intracranial haematomas as a result of spontaneous haemorrhage or trauma is of particular relevance in the event of prior intake of platelet aggregation inhibitors. We describe the effect of desmopressin acetate as a means of temporarily stabilising the platelet function. *Patients and Methods*. The platelet function was analysed in 10 patients who had received single (*N* = 4) or multiple (*N* = 6) doses of acetylsalicylic acid and 3 patients (control group) who had not taken acetylsalicylic acid. All subjects had suffered intracranial haemorrhage. Analysis was performed before, half an hour and three hours after administration of desmopressin acetate. Statistical analysis was performed by applying a level of significance of *P* ≤ 0.05. *Results*. (1) Platelet function returned to normal 30 minutes after administration of desmopressin acetate. (2) The platelet function worsened again after three hours. (3) There were no complications related to electrolytes or fluid balance. *Conclusion*. Desmopressin acetate can stabilise the platelet function in neurosurgical patients who have received acetylsalicylic acid prior to surgery without causing transfusion-related side effects or a loss of time. The effect is, however, limited and influenced by the frequency of drug intake. Further controls are needed in neurosurgical patients.

## 1. Introduction

**Figure 1:**
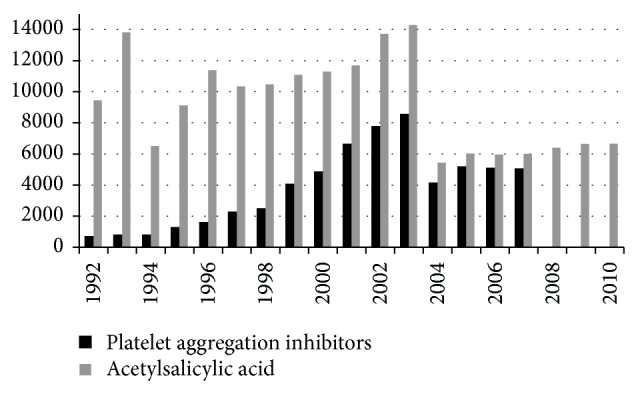
Drug prescribing: platelet aggregation inhibitor according to the German* Rote Liste* and generic drug: acetylsalicylic acid prescribed at the expense of the statutory health insurance funds [3].

Platelet aggregation inhibitors such as acetylsalicylic acid, adenosine diphosphate (ADP) receptor inhibitors, and vitamin K antagonists are a fixed constant in the treatment of acute and chronic atherothrombotic vascular diseases, including myocardial infarction, acute coronary syndrome, peripheral arterial obstructive disease, and atrial fibrillation [1, 2]. The prescribing data for platelet aggregation inhibitors and sales figures for acetylsalicylic acid suggest that their use is becoming more widespread (Figure 1) [3]. Their prescription and use are dependent on comorbidities and age, however, which can increase the attendant risk of haemorrhage [1].

These drugs, which are being used increasingly in the elderly, are of neurological importance in the event of intracranial haemorrhage, for instance. The types of intracranial haemorrhage deserving special mention are intracerebral, acute, and chronic subdural haemorrhage, and spontaneous subarachnoid haemorrhage.

**Figure 2:**
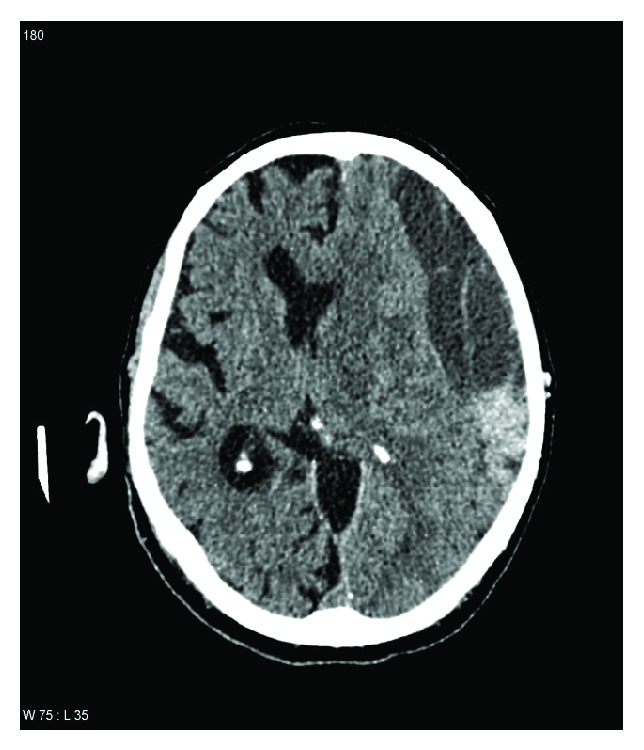
Computed tomography of a left-sided chronic subdural haematoma.

**Figure 3:**
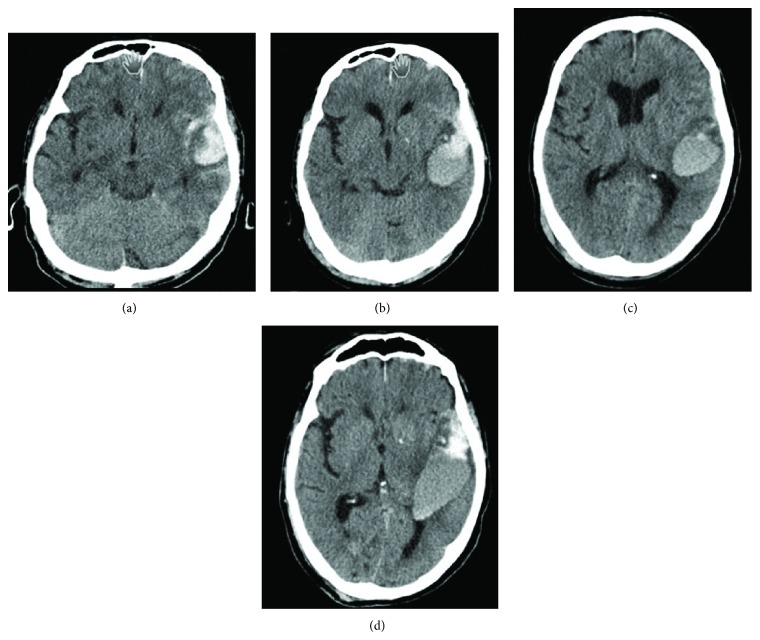
Spontaneous course of traumatic intracerebral haemorrhage with platelet aggregation inhibitor: 79-year-old patient hit by a car when walking in the city. On admission, he had a Glasgow Coma Score of 13. Findings on admission were left temporal contusion (a) which had increased in size after 24 hours (b). The patient then developed dysphasia. Although there was only a slight increase in size after a further 4 days, with the clinical condition remaining stable (c), an additional, relevant progression in size was noted on day 5 in the presence of seizures (d). Surgery was not performed since the patient never lost consciousness and did not develop severe hemiparesis.

Approximately 20% of all patients admitted with traumatic brain injury are found to have acute subdural haematoma, which primarily occurs between the age of 31 and 47 years. The mortality rate can exceed 75% [4]. Out of 100,000 patients, 10–30 suffer spontaneous intracerebral haemorrhage. The incidence of intracerebral haemorrhage associated with strokes is approximately 15%. The elderly are more likely to suffer intracerebral haemorrhage than younger individuals. The treatment outcome depends on the extent and localisation of the haemorrhage [5]. Chronic subdural haematoma (Figure 2) is one of the most common forms of traumatic intracranial haemorrhage [6] and is often caused by a mild traumatic incident only seldom recalled by the patient [7]. Common to all these types of haemorrhage is the fact that more extensive and prolonged bleeding, as well as a greater risk of secondary haemorrhaging, can be expected if the patient is already taking a platelet aggregation inhibitor [8, 9] (Figure 3).

When faced with the dilemma of prohemostatic measures for minimising the complications of intracranial haemorrhage and an indication for platelet aggregation inhibition, the question of the best possible treatment arises. Desmopressin acetate, which is known to attenuate the effect of acetylsalicylic acid, has been studied in cardiac diseases [10–14]. Desmopressin (1-deamino-8-d-arginine vasopressin) increases plasma factor levels due to the endogenous release of coagulation factor VIII (FVIII), von Willebrand factor (vWF) and tissue plasminogen activator (tPA), enhanced platelet adhesion, and reduced bleeding time [10–13, 15]. The exact mechanism of action of acetylsalicylic acid, however, has not been fully explained.

Here we discuss the possibility of attenuating the effect of acetylsalicylic acid based on an observational study in patients with intracranial haemorrhage.

## 2. Patients and Methods

The local ethics committee approved the laboratory and haematological tests in patients with traumatic brain injury and impaired consciousness.

### 2.1. Patients

Between 2008 and 2014, patients who had been admitted for acute intracranial haemorrhage and were already using acetylsalicylic acid, along with control patients, were enrolled in the study. Enrolment depended on the possibility to perform laboratory tests and so took place only during the day rather than at night and also depended on the availability of suitable patients, who were selected if they were available for analysis between 8.00 a.m. and 3.30 p.m. In addition, patients were only enrolled by three neurosurgical members of the study team.

The inclusion criteria required patients to be over 18 years of age and have a diagnosis of isolated spontaneous or traumatic intracranial haemorrhage confirmed by computed tomography. The haemorrhage alone had to be a correlate of a neurological deficit such as coma, hemiparesis, or aphasia and thus presents a surgical indication, or the progressive size had to suggest that a clinically relevant deterioration was likely (compression of basal cisterns, midline shift). A further inclusion criterion was evidence (communicated personally or in writing) of the intake of acetylsalicylic acid within 24 hours prior to admission.

The exclusion criteria comprised intake of other anticoagulants or platelet aggregation inhibitors, known coagulation disorders, alcoholism, known hypercoagulable tendency, renal failure, hypothermia (<35°C), multiple trauma, or polytrauma.

In doing so, a distinction was made between patients having taken acetylsalicylic acid once within 24 hours prior to admission (Pat_SIN_) and those having taken the drug regularly (daily) or more than once (Pat_MUL_). Patients who were conscious could be questioned; otherwise, information was taken from the records of other hospitals, documentation from previous stays at our hospital, or documentation obtained from a care facility or the general practitioner. Patient details are provided in Table 1.

The control group comprises 3 patients (one woman aged 52 years with spontaneous subarachnoid haemorrhage; one man aged 79 years with traumatic brain injury; one man aged 56 years with traumatic brain injury). Since—unlike the patients with intracranial haematoma—predominantly normal values were found initially and the values for platelet aggregation inhibition were normal, no further blood tests were performed over the course.

All patients were monitored intensively for intracranial haemorrhage and their potential (cardiac and circulatory) risks following administration of desmopressin.

### 2.2. Desmopressin Acetate and Platelet Function Tests

There are numerous recommendations to also use desmopressin acetate as a treatment for platelet function disorders or mild thrombocytopenia. Desmopressin acetate is used to improve primary haemostasis—not because of the enhanced aggregability of the platelets, but rather due to their increased adhesiveness [16].

**Figure 4:**
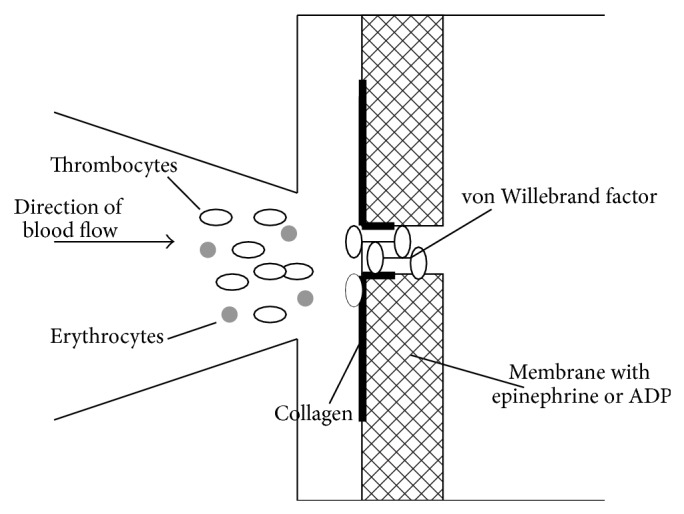
Functional principle of platelet function analysis 100 (PFA100) [17].

Platelet function was tested using platelet function analysis (PFA-100, Dade Behring, Germany), which measures the time required for a platelet plug to form and seal a semipermeable membrane. This membrane is coated in collagen. As under physiological conditions, it serves as an initial matrix for platelet adhesion in relation to the von Willebrand factor found in the tested blood. During the test, the platelets bind directly with the collagen, which in turn leads to platelet activation. Adherent platelets are degranulated on contact with the agonists' epinephrine and adenosine phosphate, which are also found in the membrane. Activation of free-flowing platelets and blood clot aggregation is the outcome. Consequently, the opening in the membrane gradually closes (Figure 4) [17]. The standard values for membrane closure range from 68 to 120 seconds for adenosine phosphate and 54 to 165 seconds for epinephrine. The uneventful use of PFA-100 in neurosurgical patients is documented in the literature [18].

### 2.3. Test Points

Platelet function was analysed prior to administration of desmopressin (T1). Desmopressin acetate was administered over a period of 30 minutes so as to avoid flushing and hypotension. The platelet function was then analysed half an hour (T2) and three hours (T3) after administration. All patients were given 24 *μ*g in 100 mL physiological saline solution over a period of 30 minutes. The time between sampling and starting the test was less than 15 minutes and did not hinder the procedures with respect to potential surgery.

Statistical analysis comprised the Wilcoxon and Mann-Whitney *U* tests, with a significance level of *P* ≤ 0.05.

## 3. Results

During the specified period, 10 patients on acetylsalicylic acid with the following conditions were enrolled: intracerebral haemorrhage (*N* = 3), cerebral infarctions (*N* = 2), traumatic brain injury with contusion or acute subdural haematoma (*N* = 2), spontaneous subarachnoid haemorrhage with hydrocephalus (*N* = 1), and chronic subdural haemorrhage (*N* = 2). Acetylsalicylic acid had been taken once by 4 patients and multiple times by 6 patients. Three patients were recruited as controls (Table 1). Seven of these 10 patients underwent surgery immediately after admission (Pat_SIN_ *N* = 3, Pat_MUL_ *N* = 4); the remaining 3 patients were monitored under intensive care conditions.

The exact dose of acetylsalicylic acid could not always be determined, especially in those patients having taken only one dose. In the others, the dose did not exceed 100 mg.

**Figure 5:**
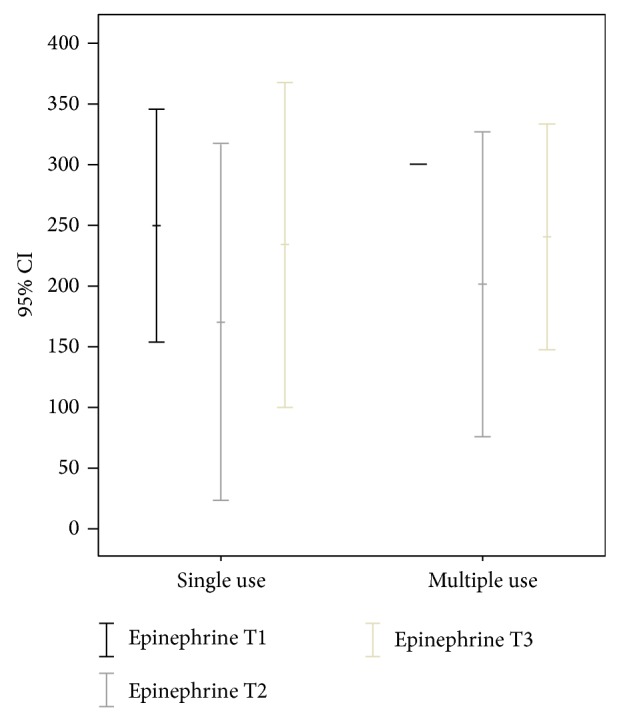
Results of platelet function analysis using epinephrine prior to administration (T1), half an hour after administration (T2), and three hours after administration (T3); *y*-axis in seconds.

**Figure 6:**
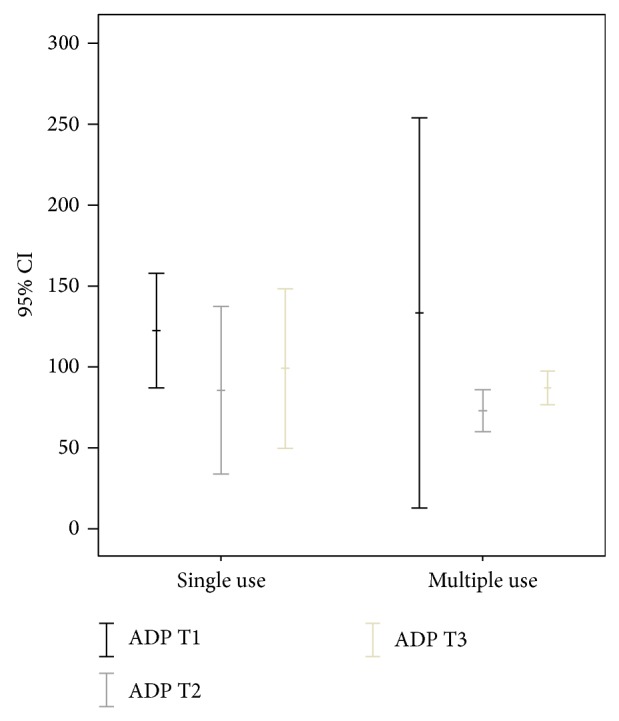
Results of platelet function analysis using adenosine phosphate (ADP) prior to administration (T1), half an hour after administration (T2), and three hours after administration (T3); *y*-axis in seconds.

1. The platelet function was found to be impaired in both patient groups (Pat_SIN_ and Pat_MUL_) at T1. Figures 5 and 6 illustrate the mean membrane occlusion times for time point T1, with results exceeding the norm of 120 seconds (adenosine phosphate) and 165 seconds (epinephrine).
  - In the adenosine phosphate samples, the mean time at T1 was 122.7 seconds (SD 21.8) for Pat_SIN_ and 126.6 seconds (SD 89.0) for Pat_MUL_ (*P* = 0.394).
  - The epinephrine samples revealed a mean occlusion time at T1 of 249.5 seconds (SD 59.94) for Pat_SIN_ (*P* = 0.068).
2. The mean value at T1 in the epinephrine tests was more than 300 seconds for Pat_MUL_ (Figure 5).
3. Platelet function had returned to normal or close to normal in both groups at T2 after administration of desmopressin acetate (Figures 5 and 6).
  - In the adenosine phosphate samples, the mean time at T2 was 86.3 seconds (SD 32.06) for Pat_SIN_ and 73.83 seconds (SD 9.45) for Pat_MUL_ (*P* = 0.831).
  - The epinephrine samples revealed a mean occlusion time at T2 of 170.5 seconds (SD 92.16) for Pat_SIN_ and 192.8 seconds (SD 92.86) for Pat_MUL_ (*P* = 0.829).
4. The platelet function was again impaired in both groups at T3, though the values were worse in the Pat_MUL_ group under epinephrine (Figures 5 and 6).
  - In the adenosine phosphate samples, the mean time at T3 was 99.5 seconds (SD 30.73) for Pat_SIN_ and 86.3 seconds (SD 7.91) for Pat_MUL_ (*P* = 0.829).
  - The epinephrine samples revealed a mean occlusion time at T3 of 234 seconds (SD 83.53) for Pat_SIN_ and 212.7 seconds (SD 94.78) for Pat_MUL_ (*P* = 0.998).
5. There were no relevant deviations from the reference values in the control group: mean ADP was 106 seconds (SD 18.5) and epinephrine 135,6 seconds (SD 16.5). There was one case of a relevant, secondary increase in intracranial haemorrhage with clinical deterioration. Surgery was not, however, indicated at any time.

No intraoperative bleeding complications or fluid or electrolyte imbalances were observed. No blood transfusions or reoperations were performed in this small cohort. The mean serum sodium and potassium levels, as well as urine volume, remained within the reference range or remained clinically irrelevant and so did not necessitate intervention.

## 4. Discussion

Taking traumatic brain injury as an example, there have been numerous reports of expansion in intracerebral haemorrhage within hours to days without the additional influence of external factors [19, 20]. Impaired coagulation in traumatic brain injuries is reported at an incidence of up to 26% [21]. Survival and outcome after traumatic brain injury depend on any expansion in the preexisting haemorrhage [20]. Haemorrhagic volume also plays an influential role in survival in the case of spontaneous intracerebral haemorrhage. The more extensive the haemorrhage, the poorer the treatment outcome when measured on the Glasgow Outcome Scale [20].

Bleeding tendency can be increased by prior treatment with acetylsalicylic acid. Desmopressin acetate is capable of significantly reducing blood loss and thus minimising the haemorrhagic risk [12, 14, 20].

As an antihemorrhagic agent with enhanced factor VIII coagulation activity, desmopressin acetate is authorised for use before surgery and tooth extraction and after traumas in mild to moderate haemophilia A and von-Willebrand-Jürgens disease [16]. However, paradigms are constantly shifting and there is still a shortage of data. There have been no reports of approval with respect to stabilising the platelet function in intracranial haemorrhage or prior to intracranial interventions following intake of acetylsalicylic acid. Aspirin has been identified as an independent predictor for death in patients suffering spontaneous intracranial haemorrhage [9].

We found that desmopressin acetate is a safe way to stabilise the platelet function in patients with intracranial haemorrhage if they are only taking acetylsalicylic acid.

We were not able to determine the exact dose of acetylsalicylic acid or the exact time between intake and administration of desmopressin acetate. However, this matter displays daily clinical life in which decisions must be done on the basis of just little information. Hence, our results must be understood within mentioned conditions.

The platelet function tended to return to normal relatively rapidly, that is, after 30 minutes, using a dose of 24 *μ*g. As a rule, this corresponds to the time it takes to admit the patient and prepare for emergency surgery. No electrolyte or fluid imbalances were found in our patient population. However, the platelet function deteriorates over the further course. Nevertheless, the question whether to administer desmopressin acetate immediately after diagnosing intracranial haemorrhage under acetylsalicylic acid or whether to only apply the drug preoperatively remains unanswered. Further studies must be conducted to verify the benefit of intranasal or intravenous administration of desmopressin acetate [22, 23].

We recommend administration of desmopressin in intracerebral haemorrhage so as to improve the coagulation status, as described in the literature (20 kg body weight = approximately 8 *μ*g, 50 kg body weight = approximately 20 *μ*g, 100 kg body weight = 40 *μ*g, or 0.3 *μ*g/kg body weight), followed by monitoring of the electrolyte and fluid balance [16, 24] in order to optimise the surgical setting.

Administration of platelet concentrates can be considered after applying desmopressin acetate; we would not recommend it, however, due to a lack of data [25–28]. Prolongation of normal platelet function is not immediately apparent as a result, and a more profound benefit would also appear doubtful given the fact that desmopressin acetate itself can stabilise the platelet function. Simultaneous administration of tranexamic acid should stabilise the blood clot, but further studies should be conducted [29].

## 5. Conclusion

Desmopressin acetate can improve the platelet function after 30 minutes in intracranial haemorrhage following acetylsalicylic acid intake. The coagulative status can thus be restored to normal levels in terms of platelet function for between 30 minutes and 3 hours. When administering desmopressin acetate in intracranial haemorrhage under acetylsalicylic acid, cardiac function, electrolyte, and fluid balance must be closely monitored.

## Figures and Tables

**Table 1a:** (a) Multiple doses

Gender	Age	Weight (kg)	Height (cm)	Cause	Sodium (prior/after)	Surgery
M	72	85	179	ICH	139/138	Yes
M	52	73	179	TBI	135/140	No
M	72	85	180	ICH	134/132	Yes
M	72	81	164	ICH	139/139	No
M	52	65	179	cSDH	138/133	Yes
M	66	89	160	CI	136/139	Yes

**Table 1b:** (b) Single dose

Gender	Age	Weight (kg)	Height (cm)	Cause	Sodium (prior/after)	Surgery
F	44	59	165	CI	137/137	Yes
M	49	80	180	SAH	132/132	Yes
M	76	70	175	TBI	128/134	No
M	80	80	170	cSDH	142/142	Yes

**Table 1c:** (c) Control group

Gender	Age	Weight (kg)	Height (cm)	Cause	Sodium	Surgery
M	79	87	174	TBI	142	No
M	56	79	176	TBI	138	No
F	52	62	168	SAH	136	Yes
